# Influence of Annealing Treatment on Microstructure and Properties of Ni-Rich NiTi Alloy Coating Prepared by Laser Cladding

**DOI:** 10.3390/ma15093298

**Published:** 2022-05-04

**Authors:** Yuqiang Feng, Ziyi Gao, Zhengfei Hu

**Affiliations:** School of Materials Science and Engineering, Tongji University, Shanghai 201804, China; yuqiang_feng@tongji.edu.cn (Y.F.); 2032923@tongji.edu.cn (Z.G.)

**Keywords:** laser cladding, NiTi alloy, heat treatment, microstructure, wear

## Abstract

NiTi alloys are widely known for their shape memory effect and super-elasticity. In this study, the laser cladding method was applied to prepare Ni-rich NiTi alloy coatings on 316L stainless steel substrate. The microstructure, phase composition, element distribution and phase transformation behavior of the coatings were investigated in as-fabricated and annealing-treated states. The results indicated that the recrystallized microstructure obtained and the content of Ni_3_Ti and Ti_2_Ni phases increased significantly with a rising annealing temperature. Annealing treatment also induced a decrease in the phase-transition enthalpy and a rise in the transformation temperature, even though no obvious martensite transformation was observed. This was suppressed due to the Fe element diffused from the substrate and was probably retarded by the mounting metallic compounds formed during annealing as well. The mechanical properties have also improved obviously; coatings annealed under 850 °C exhibited the highest microhardness of 839 HV, and the wear resistance of the coatings after annealing was enhanced with an 11% average wear mass loss reduction.

## 1. Introduction

NiTi shape memory alloy (SMA) was discovered by William J. Buehler et al. at the Naval Ordnance Laboratory (NOL) in the late 1950s, so it was also named as Nitinol [1]. In addition to the unique shape memory effects, NiTi alloys are also well known due to their super-elasticity and biocompatibility [2,3]. Since then, NiTi alloys have been widely used in aerospace, marine, military, automobile, biomedicine and other fields for applications such as antennas, connectors, actuators, cryogenic fittings, stents, tissue implants and medical devices [4,5,6]. In addition to the near equiatomic NiTi alloy (also called 55NiTi with 55 wt.% Ni), 60NiTi (with 60 wt.% Ni) is a Ni-rich version of the equiatomic NiTi alloy, which exhibits not only super-elasticity and shape memory effects, but also high tensile strength as well as excellent abrasion and corrosion resistance; therefore it is considered an ideal material for aerospace bearing applications [7,8].

However, the outstanding properties of NiTi alloys also make them difficult to work with; the manufacturing and processing of these alloys are very challenging, which limits their further extensive utilization [9,10,11]. In recent years, with the development of additive manufacturing (AM), which produces parts or components with certain shapes by adding layers of powders progressively [12], the application of AM methods has gained a lot of attention, such as laser metal deposition (LMD), selective laser melting (SLM) and laser cladding, for the manufacturing of NiTi alloy parts [13,14]. Laser cladding with NiTi alloy powders, applied as a new coating technology, has become one of the research hotspots for improving substrate surface performance. For example, Norhafzan et al. prepared NiTi cladding layers on tool steel and found that the surface hardness was almost three times that of its substrate [15]. Mokgalaka et al. studied the laser deposition of NiTi coatings for corrosion improvement and found an appreciable increase in corrosion resistance, in which the effect of the element Ti was more dominant [16]. Liu et al. investigated the microstructure of NiTi cladding layer on the TA2 (titanium alloy) substrate, which consisted of NiTi, Ni_3_Ti and NiTi_2_ intermetallic compounds [17].

Nevertheless, compared with the conventional coating methods, laser cladding involves high solidification rates and repeated heating. The rapid cooling and solidification process leads to insufficient diffusion of the elements, which causes an inhomogeneous microstructure [18]. In addition, the rapid solidification promotes formation of a supersaturated solid solution matrix, which may contain a high concentration of vacancies and accumulate to a high density of dislocations in turn [19]. In this regard, the subsequent heat treatment has been considered for improving its chemical homogeneity and phase transformation behavior of the coating [20]. According to the Ni-Ti binary phase diagram, the equiatomic NiTi can dissolve more Ni into the matrix with an increasing temperature, and, when the composition shifts to an Ni-rich area, there would be precipitates such as Ni_3_Ti, Ni_4_Ti_3_ and Ni_3_Ti_2_, in which the Ni_4_Ti_3_ and Ni_3_Ti_2_ are in the metastable phase [21,22]. These secondary phases could increase the hardness of a NiTi alloy while reducing the ductility in return [23]. Reddy et al. reported that the degree of recrystallization increased with the increase in annealing temperature, and the Ni_4_Ti_3_ metastable phase would be decomposed by the sequence of Ni_4_Ti_3_ → Ni_3_Ti_2_ → Ni_3_Ti between 500 °C and 800 °C [24,25]. The existence of Ni_4_Ti_3_ caused the formation of R-phase, which intervenes between austenite and martensite (B2 → R → B19’) [26]. Tadayyon et al. identified that an annealing NiTi alloy above the recrystallization temperature (i.e., 550–600 °C) would cause an increase in both the martensite transformation temperature and the reverse transformation temperature with an increasing annealing temperature, which improved the shape memory behavior in the meantime [22,27]. In addition, Yan et al. studied the annealing effect on a cold-worked NiTi alloy below the recrystallization temperature and revealed that the transformation temperature increased when annealed at a lower temperature (i.e., 350–450 °C) and decreased when annealed at a relatively higher temperature (i.e., 550–650 °C) [28]. Additionally, a previous study showed that the phase composition in NiTi alloys started changing when the temperature was higher than 600 °C; the proportion of Ti_2_Ni increases with a higher formation rate and becomes the dominant phase when the annealing temperature is over 1050 °C [29]. Annealing temperature also affects the properties of NiTi alloys; Zhou et al. found that the samples annealed at 400–450 °C and with a shorter annealing time achieved better corrosion resistance [30], and Tadayyon reported a decrease in both the yield strength and ultimate strength when annealing above the NiTi recrystallization temperature [22].

Therefore, the chemical composition and annealing temperatures are very crucial for modifying the microstructure and properties of NiTi alloys [31,32]. However, current research focuses more on the equiatomic and as-cast NiTi alloy; only a few studies have investigated the influence of heat treatment on Ni-rich alloys and its application in coating. In this study, 55NiTi alloy powder and pure Ni powder are mixed according to a certain weight ratio to replace the customized 60NiTi alloy powder. While the production of this is time and labor consuming, the use of an elemental powder blend (called 55NiTi+5Ni) for laser cladding is an alternative solution that can yield a material with the required composition. The objective of this paper is to study the influence of annealing treatment on the Ni-rich NiTi alloy coatings prepared by laser cladding. The microstructure, phase composition, element distribution, phase transformation behavior and mechanical properties of the coatings were evaluated in the as-fabricated and heat-treated states. The research results provide a reference for the behavior of an annealed Ni-rich NiTi alloy and contribute to expand its potential utilization in industrial fields.

## 2. Materials and Methods

### 2.1. Material and Sample Preparation

Pure Ni powder and 55NiTi alloy powder (99.9% purity, Jiangsu Willari New Material Technology Co., Ltd., Xuzhou, China) with spherical morphology and a particle size distribution of d10 = 55.25 μm, d50 = 85.73 μm and d90 = 125.90 μm were used as feedstock materials for laser cladding. The chemical composition of the applied powders and substrate (AISI 316L stainless steel, with dimension size of 100 mm × 100 mm × 10 mm) are shown in Table 1 and Table 2, respectively, in which Bal. means balance element. NiTi alloy powder and pure Ni powder are mixed according to the weight proportion of 8:1 at 250 rpm by a planetary ball mill (QM-3SP4, Nanda Instrument, Nanjing, China) for 8 h and dried in an oven under 110 °C, while research shows that pre-alloyed powders are beneficial for a more homogeneous microstructure and stable mechanical properties [33].

**Figure 1:**
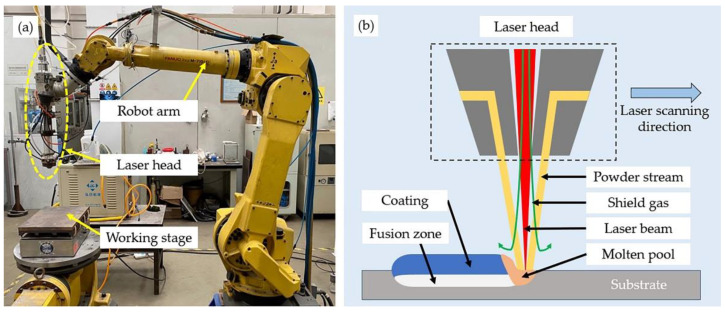
IPG YLS-10000 laser equipment: (**a**) overview of the equipment; (**b**) schematic diagram of the laser cladding process.

A high-power fiber laser (YLS-10000, IPG Photonics, Oxford, MA, USA), as shown in Figure 1, was employed for laser cladding.

The diameter of the laser beam spot was fixed by 7.2 mm with a wavelength of 1080 nm. Based on previous studies [34,35], the parameters for coating fabrication were under: 2.0 kW power, 2 mm/s laser scanning speed, 50 rpm powder feeding rate and 55% overlap ratio. Argon (99.9 vol.%) with a flow rate of 5 L/min was applied as shielding gas to prevent oxidation of the molten pool and powders. Apart from that, it was used as a powder feeding gas with a flow rate of 8 L/min as well. The substrate was preheated to 250 °C to release internal stress before laser cladding. After laser cladding, the substrate with coating was cut into numerous cuboid samples with dimensions of 5 mm × 5 mm × 10 mm for further investigation. Samples for the subsequent microstructure observations and hardness test were cut perpendicular to the laser scanning direction. These cross-section samples were grinded, polished and etched by an HF:HNO_3_:H_2_O solution with a dilution of 1:4:5. Samples for differential scanning calorimetry (DSC) tests were cut in the form of a cylinder with a height of 2 mm, a diameter of 3 mm and an average weight about 50 mg. These samples were polished to remove surface impurities.

### 2.2. Experimental Procedure

**Figure 2:**
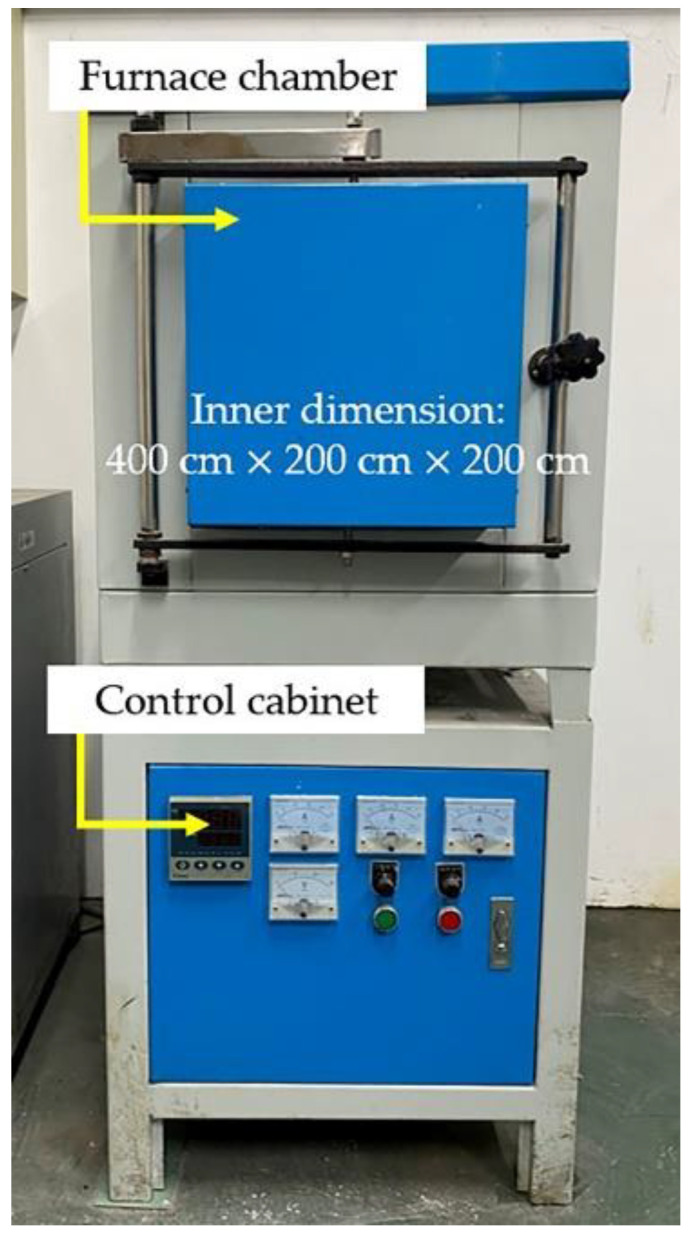
YFX-16 electrical resistance furnace used for heat treatment.

The heat treatment of the prepared cuboid samples was conducted with the use of an electrical resistance furnace (YFX16, Shanghai Y-FENG Electrical Furnace Co., Ltd, Shanghai, China), as shown in Figure 2. With a heating rate of 10 °C/min, the furnace was always heated 10 °C higher than the desired temperature, so that its temperature was allowed to drop to the designated temperature when the furnace chamber was opened later on to put in the aforementioned samples. A timer would be activated once the furnace temperature or hold time reached the set value, and subsequently, the related samples were taken out of the furnace and air-cooled under room temperature. As shown in Table 3, there were 4 groups of samples, which were annealed separately according to the different temperatures and holding time, and each group had 4 pieces to check their repeatability. In addition, HT is defined as the abbreviation of heat treatment in this article.

### 2.3. Sample Characterization

In order to investigate the microstructure of the samples before and after heat treatment, an optical microscope (OM, GX51, Olympus, Tokyo, Japan) and a scanning electron microscope (SEM, Zeiss Gemini 300, Oberkochen, Germany) with a configured energy-dispersive spectroscopy (EDS, OXFORD Xplore, Oxford, England) were used for observation. In addition, X-ray diffraction (XRD, Bruker D8 ADVANCE, Karlsruhe, Germany) was performed to study the phase composition of the coating with Cu Kα radiation at 40 kV and 100 mA and a scanning speed of 2°/min, ranging from 10° to 80°. The differential scanning calorimetry (DSC250, TA Instruments, New Castle, DE, USA) with a measuring range from −180 °C to 725 °C, temperature accuracy of 0.05 °C and enthalpy precision of 0.08%, was used to study the coating phase transformation behavior. The DSC test refers to ASTM Standard F2004-17 and was under a nitrogen atmosphere with temperature scanning ranges from −80 °C to 180 °C and a heating and cooling speed of 10 °C/min. For the friction and wear test, the experiment was conducted in a ball-on-plate configuration (Ht-1000, ZKKH Science and Technology Development Co., Ltd., Lanzhou, China) using 5 mm diameter Si_3_N_4_ balls at room temperature under a load of 15 N and a rotation speed of 224 r/min. Furthermore, a microhardness tester (HVS1000A, HuaYin Testing Machine Technology Co., Ltd., Laizhou, China) was employed in the experiment with a load of 1.961 N and a holding time of 15 s. Hardness was measured from the coating surface to the substrate at an interval of 0.2 mm and repeated 3 times at the same depth to calculate the average value.

## 3. Results and Discussions

### 3.1. Coating Phase Composition Analysis

**Figure 3:**
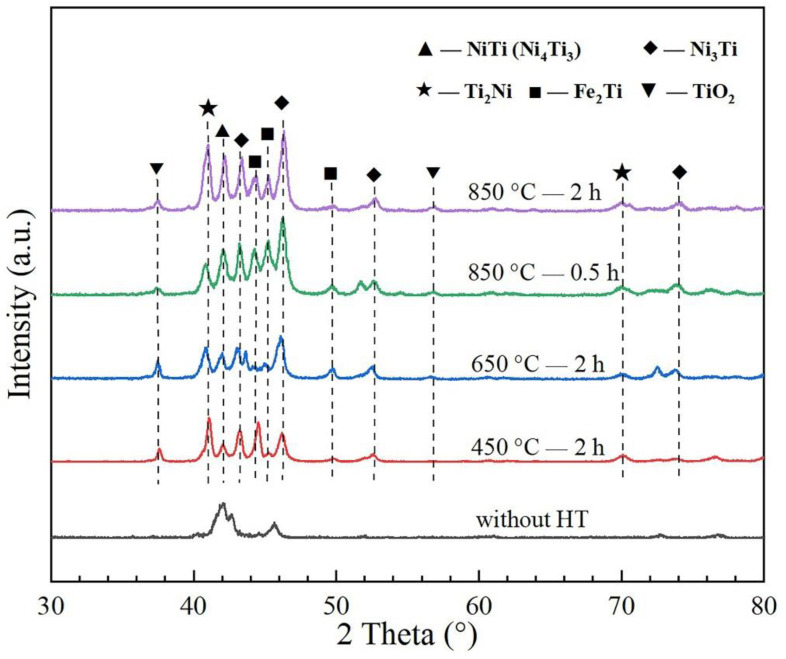
XRD patterns of the as-fabricated and heat-treated 55NiTi+5Ni coating samples at different annealing temperatures.

The analysis of coating phase composition is based on the XRD results. Figure 3 shows the XRD patterns of the as-fabricated and heat-treated 55NiTi+5Ni laser cladding samples, which has been normalized according to a standard intensity scale.

It can be seen that the coating was mainly composed of NiTi and Ni_3_Ti phase, which show the strongest diffraction peaks. In addition to that, secondary phases such as TiO_2_, Fe_2_Ti and Ni_4_Ti_3_ were also detected with a relative weak diffraction intensity after the annealing treatment. Of these, Ni_4_Ti_3_ is a metastable phase with nanoscale structure, which has similar diffraction peaks to NiTi phase. The formation of Fe_2_Ti was due to diffusion of the Fe atom from the substrate, and TiO_2_ was attributed to the oxidation of the coating surface, which has not been observed before annealing. It is worth noting that, compared with the patterns before annealing, the intensities of the characteristic diffraction peak of Ni_3_Ti (2θ = 46.53°) and Ti_2_Ni (2θ = 41.56°) are getting obviously stronger. This was probably caused by the decomposition of NiTi and Ni_4_Ti_3_ phase (NiTi / Ni_4_Ti_3_ → Ni_3_Ti + Ti_2_Ni), which were unstable when heated to a higher temperature [36,37]. In addition, the content of Fe_2_Ti increased significantly when annealing at a higher temperature, which was due to more active diffusion activity.

### 3.2. Coating Microstructural Analysis

**Figure 4:**
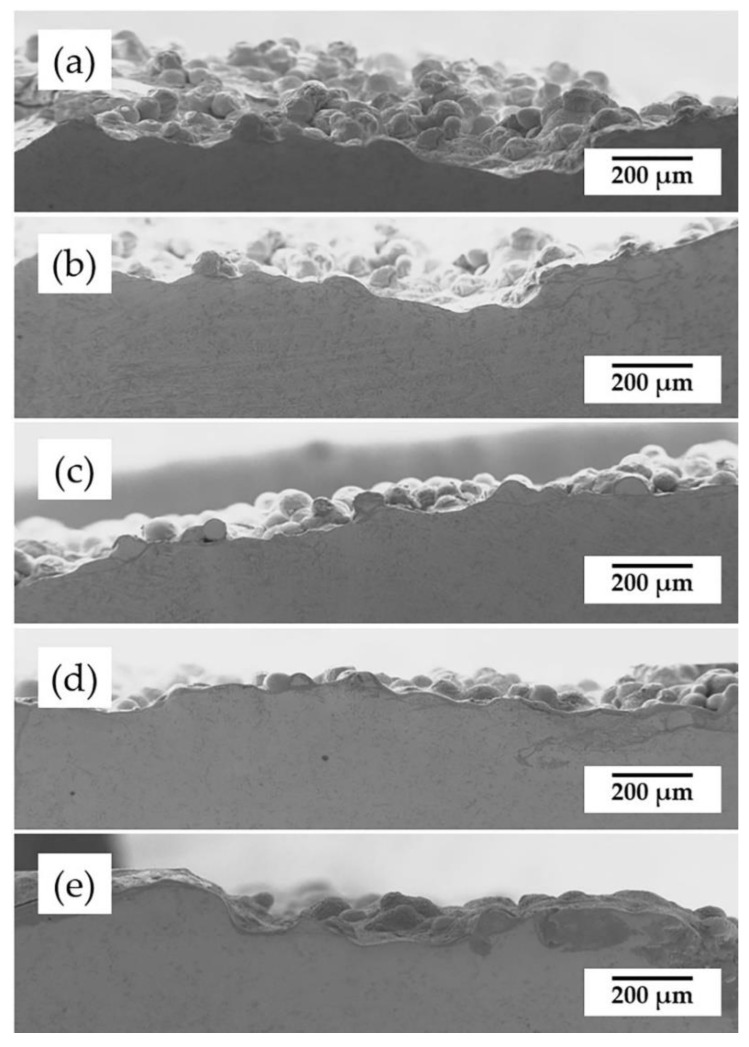
Surface morphology of the coatings before and after annealing treatment: (**a**) before annealing; (**b**) 450 °C for 2 h; (**c**) 650 °C for 2 h; (**d**) 850 °C for 0.5 h; (**e**) 850 °C for 2 h.

The surface morphology of the coatings before and after the annealing treatment is shown in Figure 4. A few unfused powders are found on the surface of the coating; while laser cladding is a quick heating and cooling process, the temperature gradient becomes larger from the molten pool to the upper surface, and therefore, these powders could not absorb the energy sufficient to melt. However, with an increasing annealing temperature, the surface flatness shows the trend improving. It is worth noting that the grain boundaries are more obviously seen in the samples annealing at 450 °C and 650 °C; however, they are no more visible when annealing at 850 °C, which is due to the occurrence of recrystallization.

**Figure 5:**
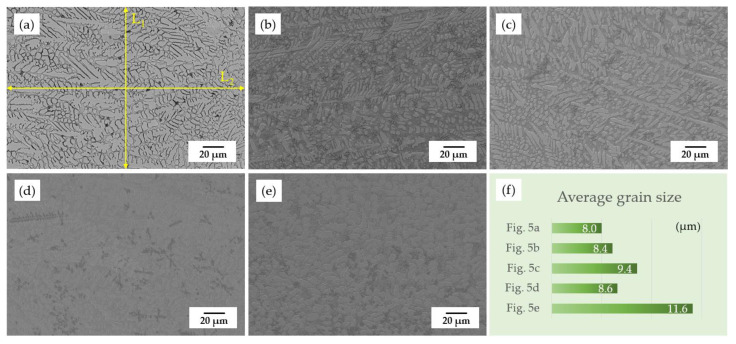
SEM morphology of the coating cross-sections before and after annealing treatment: (**a**) before annealing; (**b**) 450 °C for 2 h; (**c**) 650 °C for 2 h; (**d**) 850 °C for 0.5 h; (**e**) 850 °C for 2 h; (**f**) average grain size of the above coatings.

Figure 5 displays the SEM morphology of the coating cross-sections before and after the annealing treatment. Studies reported that the recrystallization temperature of 55NiTi alloy is between 550 °C and 650 °C and would further increase with the rising content of Ni in the alloy [22,27]. As the typical dendrite structure still remains in the coating after annealing under 650 °C, which is shown in Figure 5c, it can be shown that the recrystallization temperature of the 55NiTi+5Ni coating is at least higher than 650 °C. Figure 5d,e show the microstructure of coating after annealing under 850 °C, which displays only large single grains with black inclusions and indicates that the recrystallization has been completed. In addition, the average grain size of the coating displays an enlargement trend with an increasing annealing temperature. According to ASTM E112, the linear intercept method was applied to measure the grain size. The grain intercept count (GIC) stands for the number of times a test line cut through the grains, in which tangent hits and cutting lines that end within a grain are considered to be a 0.5 interception. As remarked in Figure 5a, L_1_ and L_2_ are the intercept lines, and the grain size is calculated through the following formula:

(1) $$d=\frac{\begin{array}{c} L_{1}/GIC_{1}+L_{2}/GIC_{2} \end{array}}{2}$$

The measuring results are listed in Table 4, and Figure 5f is the bar chart of the results. It can be seen that the coating average grain size became larger with an increasing annealing temperature and a prolonged holding time as well.

**Figure 6:**
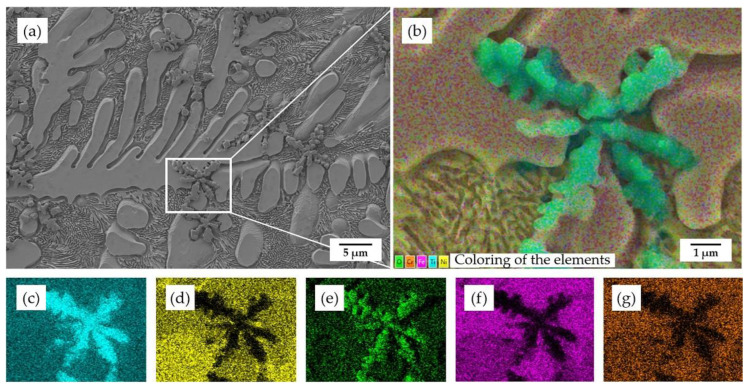
EDS regional scanning results of the coating after annealing under 450 °C: (**a**) original SEM morphology; (**b**) EDS composite image; (**c**) element Ti; (**d**) element Ni; (**e**) element O; (**f**) element Fe; (**g**) element Cr.

In comparison to the coating without heat treatment, there are more black inclusions found in the heat-treated coatings, which occur among the dendrites and inter-dendrites area. According to the EDS regional scanning results of these inclusions, shown in Figure 6, it can be concluded that the black inclusions are titanium oxide.

As shown in Figure 5b, it is noticeable that there is a lot of titanium oxide dispersed within the dendrites when samples annealed at 450 °C. However, when samples are annealed at higher temperatures, there is less dispersion of titanium oxide observed. On the one hand, due to the increasing temperature, the gap among the grains becomes smaller, which leads to the escape of oxides originally stored in the gaps. On the other hand, due to the more active diffusion activities at higher temperatures, more Fe atoms from the substrate diffused to the coating, which caused the formation of Fe_2_Ti and partly consumed the titanium atoms.

**Figure 7:**
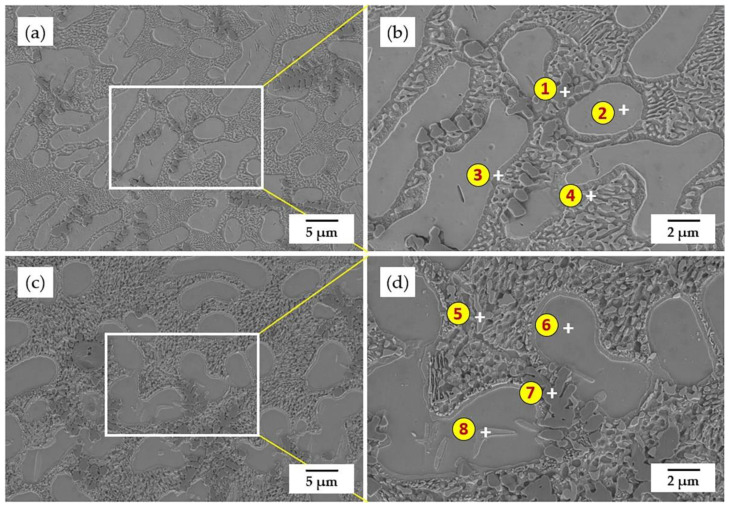
SEM morphology of the coating after annealing under 850 °C: (**a**,**b**) keep 0.5 h; (**c**,**d**) keep 2 h.

Figure 7 shows the SEM morphology of the coating after annealing under 850 °C.

It is worth noting that the average grain size of the coating with a holding time of 2 h is larger than that with a holding time of 0.5 h, which explains that, with the extension of the holding time, the grains would further grow after recrystallization [38]. In order to identify the relationship between the coating microstructure and phase composition, EDS was employed to analyze the element distribution within the phases, and the scanning results are shown in Table 5. Based on the EDS results of Position 2 and Position 6, marked in Figure 7, their atomic ratio of Ni and Ti is close to 1:1 so that the separate large grain can be assigned as NiTi phase. In contrast, Position 3 is rich in Ni with a Ni-Ti atomic ratio of about 3:1, which can be identified as Ni_3_Ti. With the diffusion of Fe atoms, which participated in the nucleation process during recrystallization of the coating, Fe_2_Ti phase was formed and precipitated from the Ni_3_Ti phase, where Position 4 and Position 5 were occupied, and the fraction of regions with an eutectic microstructure of Fe_2_Ti and Ni_3_Ti increased due to an accelerated Fe diffusion into the coating, especially under higher temperatures and extended holding time. In addition, the formation of Fe_2_Ti led to an increase in the atomic ratio of Ni to Ti in the coating, further promoting the precipitation of Ni_3_Ti intermetallic compound, which is more stable and has a higher melting point. Based on the EDS result of Position 8, it seemed to be Ni_4_Ti_3_. However, Ni_4_Ti_3_ phase is a metastable phase, which would decompose at higher temperatures and with prolonged exposure. In addition to that, Ni_4_Ti_3_ has a typical lenticular nanoscale shape [39,40]. Therefore, it was assumed to be the start phase of the decomposition inside the NiTi phase, which needs further TEM observation. Meanwhile, a small amount of Cr atoms, which diffused from the substrate, were detected within the NiTi and Ni_3_Ti phase. According to the site preference rule of ternary alloys to NiTi, Cr atoms are more likely to replace the Ti atoms in the crystal structure, since Cr has similar atom radius and electronegativity to a Ti atom [41].

### 3.3. Coating Martensite Transformation Behavior

**Figure 8:**
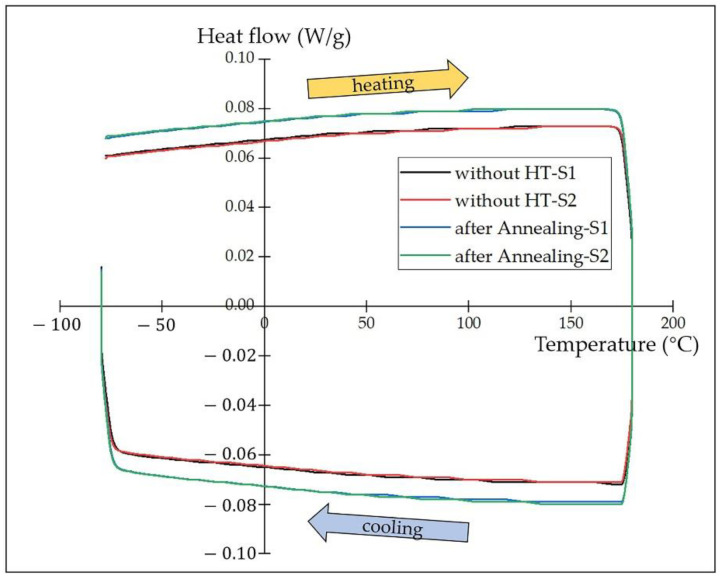
DSC curves of 55NiTi+5Ni coating before and after annealing under 850 °C.

Figure 8 shows the DSC curves of the 55NiTi+5Ni coating before and after annealing treatment. However, it looks different from the main publication: there is no obvious absorption peak detected during the whole heating and cooling cycle, but this does not mean that the martensite transformation has not taken place in the measuring temperature range. Table 5 shows that a few Fe atoms are detected in the NiTi phase, which substituted the Ni atoms in the B2 structure [41]. Previous studies reported that the addition of Fe has influence on the phase transformation behavior of NiTi alloys, which could suppress the first-order martensite transformation and reserve transformation. In addition, the second-order-like phase transformation from an incommensurate state to a commensurate state might take place [42,43]. In addition to that, according to the XRD results, there were more secondary phases, such as Ni_3_Ti, Ti_2_Ni and Fe_2_Ti, precipitated after annealing. The second-order-like martensite transformation might be retarded by the formation of these metallic compounds as well. Meanwhile, previous research reported that the martensite transformation temperature of the NiTi alloy decreased with the increasing content of Ni [44]. With the decomposition of NiTi into the more stable Ni_3_Ti and Ti_2_Ni by higher annealing temperatures, the content of Ni in the NiTi phase decreased. Therefore, it is supposed that the martensite transformation temperature of the coating would increase with an increase in the annealing temperature. Apart from that, the compensated heat flow for the heat-treated samples was about 0.007 W/g higher than the samples without annealing during both the heating and cooling process, which indicates that the heat-treated coating samples consumed more energy.

**Figure 9:**
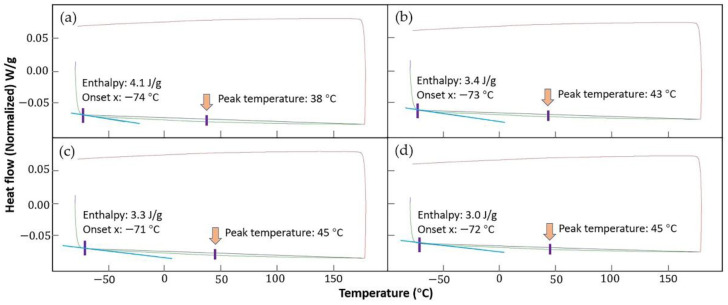
Integral calculation of the phase-transition enthalpy with TA Instruments software TRIOS based on the coating DSC curves: (**a**,**b**) before annealing; (**c**,**d**) after annealing.

As there was no obvious phase transformation observed during the DSC test, the transformation temperatures and phase-transition enthalpy were assessed with TA Instruments software TRIOS. Figure 9 shows the integral calculation process based on the coating DSC curves; the transformation temperatures and heat flows were determined by the intersections of a baseline and the tangents to a thermal peak in software, and the results are listed in Table 6. The phase-transition enthalpy is closely related to the progress of the phase transformation, which is accompanied by the release or absorption of heat. The phase-transition enthalpy of the coatings after annealing is lower than that without heat treatment, which indicates that the martensite transformation is more likely to take place in the coating without annealing. In addition, the calculation result proves that the phase transformation temperature increased after the annealing treatment.

### 3.4. Coating Properties Analysis

#### 3.4.1. Coating Surface Hardness

**Figure 10:**
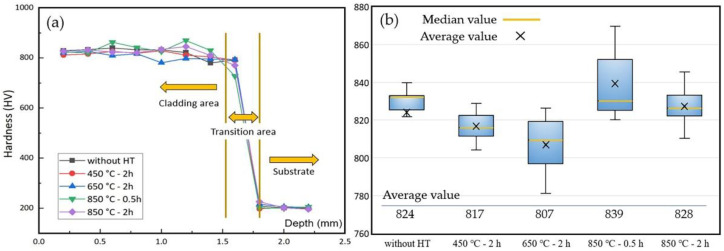
Coating microhardness test results: (**a**) hardness along the depth; (**b**) hardness boxplot of the coating cladding area.

Figure 10a displays the microhardness test results for the coatings along the depth, which could be clearly divided in three areas: coating cladding area, coating-substrate transition area and substrate, respectively. The microhardness distribution was generally consistent from sample to sample, indicating that the samples were fairly homogeneous over the areas measured. Figure 10b shows a hardness boxplot of the coating cladding area, the region that bears load and friction in the coating. The hardness of the coating decreased when annealed at lower temperatures (e.g., 450 °C and 650 °C), which is due to a decrease in the defect density in the coating with the release of internal stress [45]. However, coatings annealed under 850 °C exhibit a recovery of the hardness, which is even a little bit higher than samples without heat treatment. The phase segregation of Ni_3_Ti and Ti_2_Ni causes precipitation hardening, which can be attributed to the combined effect of crystallization, nucleation and grain growth [46]. This conclusion is also supported by the XRD, SEM and EDS results mentioned above.

#### 3.4.2. Coating Wear Resistance

**Figure 11:**
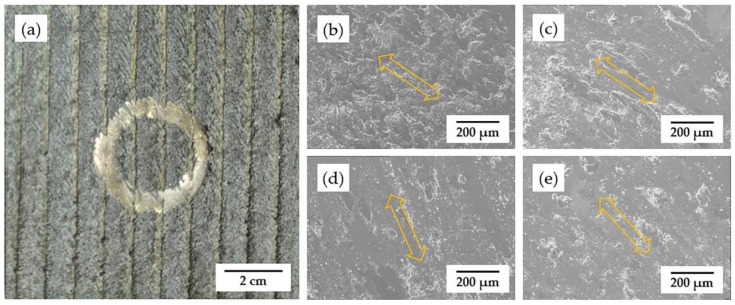
Surface morphology of the coatings after friction and wear test: (**a**) overview; (**b**) without HT; (**c**) 450 °C for 2 h; (**d**) 650 °C for 2 h; (**e**) 850 °C for 2 h.

**Figure 12:**
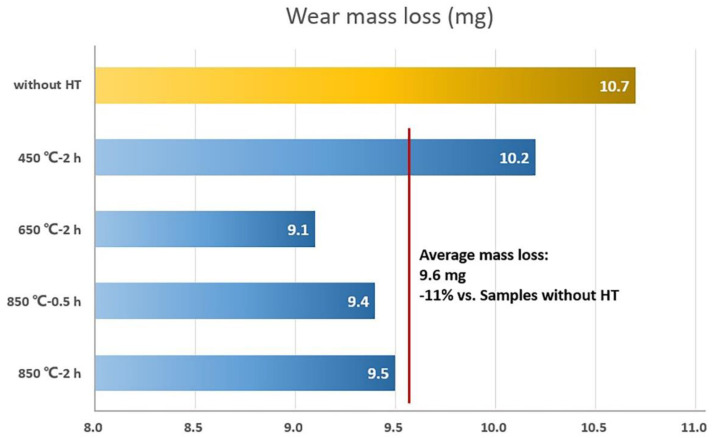
Wear mass loss results for the coatings during the friction and wear test.

The surface morphologies of coatings after the friction and wear test are shown in Figure 11, and the sliding directions, marked with arrows, are clear to recognize. It is obvious to see that, coating without annealing shows more wear debris on the surface, which is due to the poor surface condition with some unfused powders, as shown in Figure 4a. Figure 11c–e are SEM surface morphologies of the coatings after annealing, which exhibit less wear debris instead. In addition, the coating surface without heat treatment is supposed more brittle, while the surface cooled in the air within a very short time after the high-power laser beam moved forwards. Nevertheless, all surface morphologies share a similar wear appearance but with different levels of severity, indicating the same wear mechanism during the friction and wear test. This is considered delamination wear. Figure 12 displays the results of wear mass loss of the coatings during the friction and wear test. The coatings after annealing under 650 °C and 850 °C show obviously less wear mass loss, which might be attributed to an improvement in the ductility after recrystallization. Generally, with annealing treatment, the wear resistance of the coatings has been significantly improved with an 11% average mass loss reduction.

## 4. Conclusions

This paper investigates the influence of annealing treatment on the microstructure, phase composition, transformation behavior and mechanical properties of 55NiTi+5Ni coating prepared via laser cladding. Based on the obtained results and analysis, the following main conclusions can be made:

1. The untreated coating mainly consists of NiTi and Ni_3_Ti phases with a typical dendrite structure. With the annealing temperature rising, the content of Ni_3_Ti and Ti_2_Ni visibly increased.
2. The coating microstructure transformed from dendrite structure to single grains because of recrystallization, and the recrystallization temperature of the 55NiTi+5Ni coating is assumed to be between 650 °C and 850 °C.
3. There is no martensite transformation observed in the DSC test. The martensite transformation could be suppressed by Fe atoms diffused from the substrate and probably retarded by the numerous metallic compounds after annealing as well. However, the software calculation results show that the phase-transition enthalpy decreased and the transformation temperature rose after annealing.
4. The coating annealed under 850 °C shows the highest microhardness due to the precipitation hardening, and the wear resistance was also improved after an annealing treatment with an 11% average wear mass loss.

The research provides a reference for using blend powder to replace the rare 60NiTi alloy powder in the preparation of Ni-rich NiTi alloy coating and proved the possibility of further improving the coating performance via annealing treatment. However, since the phase transformation temperature of the Ni-rich NiTi alloy is very sensitive to the content of Ni and the addition of Fe in the alloy, additional DSC tests are needed for further investigation.

## Figures and Tables

**Table 1:** Chemical composition of Ni and 55NiTi alloy powders (wt.%).

Powders	Ni	Ti	Fe	Nb	Co	C	Si	O
Pure Ni	Bal.	-	0.003	-	0.020	0.020	0.003	0.006
55NiTi alloy	56.46	Bal.	0.005	0.010	0.005	0.005	-	0.037

**Table 2:** Chemical composition of AISI 316L stainless steel substrate (wt.%).

Fe	Cr	Ni	Mo	Mn	Si	P	C	S
Bal.	16.32	10.12	2.04	0.92	0.34	0.026	0.016	0.015

**Table 3:** Heat treatment parameters for samples.

Samples	Annealing Temperature (°C)	Holding Time (min)
Group 1	450	120
Group 2	650	120
Group 3	850	30
Group 4	850	120

**Table 4:** Measuring results of average grain size with linear intercept method.

Coatings	GIC_1_	GIC_2_	Intercept Length (μm)	Average Grain Size (μm)
Figure 5a	19	28.5	L1 = 153.8 L2 = 225.1	8.00
Figure 5b	18	27		8.44
Figure 5c	19	21		9.41
Figure 5d	20.5	23		8.64
Figure 5e	16	16.5		11.63

**Table 5:** EDS scanning results of the positions marked in Figure 7.

Coating	Position	Ni (at.%)	Ti (at.%)	Fe (at.%)	Cr (at.%)	O (at.%)	Potential Phase
850 °C for 0.5 h	1	0.7	38.1	0.7	0.3	60.2	TiO_2_
	2	37.5	39.2	11.8	8.6	2.9	NiTi
	3	50.5	27.0	15.3	6.5	0.7	Ni_3_Ti
	4	40.1	26.9	23.5	7.4	2.1	Ni_3_Ti+Fe_2_Ti
850 °C for 2 h	5	43.3	21.9	24.7	6.9	3.2	Ni_3_Ti+Fe_2_Ti
	6	37.0	34.1	17.8	7.3	3.8	NiTi
	7	0.7	35.2	0.7	0.4	63.0	TiO_2_
	8	48.0	36.2	8.3	6.1	1.4	NiTi+Ni_3_Ti

**Table 6:** Calculation results of the phase-transition enthalpy and temperature of the coatings before and after annealing.

Coating	Onset Point (°C)	Peak Temperature (°C)	Normalized Enthalpy (J/g)
without HT-S1	−74	38	4.1
without HT-S2	−73	43	3.4
after annealing-S1	−71	45	3.3
after annealing-S2	−72	45	3.0

## Data Availability

Not applicable.
